# A Case of Lumbosacral Radiculoplexus Neuropathy: A Rare Complication of Diabetes Mellitus

**DOI:** 10.7759/cureus.75005

**Published:** 2024-12-02

**Authors:** Chukwudi Agogbua, Benjamine Ajoku, Ayan Mohamed, Oluwaseun Oloyede, Joy Aikpitanyi

**Affiliations:** 1 Geriatrics, Queen's Hospital, Barking, Havering and Redbridge University Hospitals NHS Trust, Romford, GBR; 2 Internal Medicine, Barking, Havering and Redbridge University Hospitals NHS Trust, London, GBR; 3 Internal Medicine, Queen's Hospital, Barking, Havering and Redbridge University Hospitals NHS Trust, Romford, GBR

**Keywords:** asymmetric, axonal degeneration, diabetic neuropathic pain, fall assessment, sensory motor neuropathy

## Abstract

Diabetic lumbosacral radiculoplexus neuropathy, also known as diabetic lumbosacral plexopathy or diabetic amyotrophy, is a rare complication of diabetes mellitus. Due to its varied clinical presentation and wide differential, it may pose a diagnostic quandary in assessing patients with proximal asymmetrical lower limb weakness. We present the case of a 74-year-old female patient with a recent onset of falls and aim to discuss the aetiology, differentials, and treatment modalities in diabetic plexopathy.

## Introduction

Lumbosacral plexopathy is a spectrum of peripheral nerve disorders primarily involving the lumbosacral plexus and spinal roots [1]. It is an uncommon condition, affecting approximately 1% of people with diabetes and highlights the importance of careful history and neurological assessment in the evaluation of patients who present with proximal lower limb weakness and pain [2]. It is often characterised by an injury to the lumbosacral plexus resulting in many symptoms with varying degrees of severity and duration [3]. Due to its proximity to various abdominal and pelvic organs, several injuries may lead to lumbosacral plexopathy as seen in trauma, hematomas, localised infections or abscesses and even scarring from previous abdominal surgeries [3]. The most common presenting complaint is pain which may be localised at the lower back [3].

In diabetic lumbosacral plexopathy, pain is usually noted at the unilateral lower limb and may be associated with paresthesia or numbness [3]. Sensory disturbances may follow a dermatomal pattern if nerve roots are affected. An altered sensation of the medial or anterior thigh may be more indicative of lumbar plexus pathology [3]. In severe cases, evidence of muscle weakness and wasting can be found in the clinical examination [3]. Due to its wide differential diagnosis, lumbosacral plexopathy may often be misdiagnosed. Differential diagnoses may include but are not exclusive to autoimmune, radiation-induced, neoplastic, traumatic, inflammatory, ischemic, or idiopathic causes [3]. Diabetes mellitus is recognised as a metabolic or inflammatory cause of lumbosacral plexopathy [3]. Although incompletely understood, there are associations with inflammation and immune-mediated microvasculitis leading to peripheral nerve injury, nerve root injury and axonal degeneration with demyelination [2]. 

We present the case of a 74-year-old woman who presented to the Emergency Department following a series of falls at home. The neurological examination raised suspicion of neuropathy as the mechanism of the patient's initial presentation to the hospital. Differentials such as diabetic neuropathy and possible cord compression were considered. Neuroimaging ruled out sinister neurological findings. Nerve conduction studies (NCSs) revealed findings in keeping with widespread sensorimotor disease with higher severity of proximal asymmetric neuropathy at the right lower limb.

Further imaging and laboratory tests were obviated to rule out other likely causes for these findings. Upon exclusion of other likely common aetiologies, it was deemed that the driving mechanism for the patient's presentation was diabetes. Patients with this condition may have good glycemic control or even a new diagnosis of diabetes before their initial presentation [2]. The patient in this case was noted to have an improving HbA1c over the year. This was the patient's first documented presentation with neuropathy. We would like to use this case to highlight the importance of a comprehensive neurological assessment of all patients who present to the hospital with falls.

## Case presentation

A 74-year-old woman presented to the Emergency Department with a three-week history of right lower limb weakness and pain as well as falls at home. Her past medical history includes type 2 diabetes (diagnosed in 1995), hypertension, and cervical intraepithelial neoplasia (excised, 2008). She had no previous surgeries or radiotherapy. She is a non-smoker. She was on Ramipril 10mg once daily and Nifedipine 5mg once daily for her anti-hypertensive management. She was on a regimen of metformin 1 gram twice daily, sitagliptin 100mg once daily and gliclazide 80mg twice daily for her anti-diabetic management. She described a deterioration in her mobility, requiring a stick. Three weeks before her initial hospital presentation, she began to experience thigh pain, weakness in her lower limbs and falls. Unfortunately, she became housebound due to a fear of experiencing further falls and feeling unsafe. The patient presented due to concerns about falling and a resulting detriment to her quality of life.

The patient also reported an unquantifiable amount of weight loss which, though significant to her, was intentional and attributed to a healthy diet. She denied any associated cardiac, respiratory, or gastrointestinal symptoms. On further questioning, the patient’s chief complaint was worsening weakness which had begun to impair her mobility. She noted right thigh pain which was burning in character and did not radiate from any other anatomical regions as described by the patient. She denied any changes to bladder and bowel continence. A neurological examination was performed by the admitting medical team before a neurology consult. 

According to the Medical Research Council grading system, muscle strength of the hips and lower limbs was assessed as follows: hip flexion right 3/5, left 5/5; hip extension right 3/5, left 5/5; hip abduction right 5/5, left 5/5; hip adduction right 5/5, left 5/5; knee flexion right 4/5, left 5/5; knee extension right 4/5, left 5/5; ankle dorsiflexion 4/5, left 5/5. Examination also revealed hyporeflexia of both knee and ankle reflexes on the right side of the lower extremities with diminished sensation in a stocking pattern bilaterally up to the knee joints. The rest of the neurological examination was unremarkable. Other aspects of the clinical examination were unremarkable. This patient reported no back or groin pain at the time. 

An MRI of the whole spine returned normal signal intensity throughout the vertebra, spinal cord, and intervertebral discs. There were degenerative changes in C3-4 and C6-7 with bilateral neural foraminal narrowing with both exiting L3 nerve roots. Bilateral neural foraminal narrowing was also noted at the exit of the left L5 nerve root. However, there was no significant central spinal canal stenosis. The CT of the chest, abdomen and pelvis was unremarkable. An MRI of the head was unremarkable. Subsequently, NCSs were arranged for the patient. Electromyography revealed acute denervation potentials in the right iliopsoas superimposed on only a few voluntary motor units (Table 1). The right iliopsoas showed acute-on-severe chronic neurogenic changes. There were moderate chronic neurogenic changes in the right tibialis anterior, right gastrocnemius and left tibialis anterior.

**Table 1:** Electromyography (EMG) study MUAP: motor unit action potential; PSW: positive sharp waves; Poly: polyphasic

		Spontaneous		Voluntary			
		Fib	PSW	Amp	Dur	Poly	IP
Muscle	Interpretation						
R.biceps	Normal	0/10	0/10	normal	normal	+	
R. Gastroc caput med	Mod. Inactive neur	0/10	0/10	++	++	++	--
R. iliopsoas	Pron subacute neur	8/10	9/10	-	++	++	--
R. inteross dors	Slight inactive neur	0/10	0/10	+	+	+	
R. Rectus femoris	Complete denervation	10/10	10/10	No voluntary MUAPs			
R. Tibialis anterior	Mod subacute neur	8/10	9/10	++	++	++	--
R. Vastus med	Complete denervation	6/10	8/10	No voluntary MUAPs			
L. iliopsoas	Normal	0/10	0/10	normal	normal	normal	
L. Rectus femoris	Normal	0/10	0/10	normal	normal	normal	
L. Tibialis anterior	Mod inactive neur	0/10	0/10	++	++	++	-

There were absent sensory responses in the distal lower limbs except for a small superficial peroneal sensory action potential (Table 2). Posterior tibial motor responses are absent bilaterally with reduced common peroneal motor responses on both sides (Table 3). Right superficial radial sensory responses were small in amplitude with absent right median sensory responses. The study showed evidence of severe, generalised axonal, sensory-motor peripheral neuropathy affecting lower limbs more than upper limbs. These findings suggest a superimposed lumbosacral plexopathy with signs of active ongoing motor axonal loss in the right lower limb. 

**Table 2:** Sensory nerve conduction study

Sensory	Latency to onset (msec)		Amplitude P-P (V)		Conduction velocity (m/s)	
	left	right	left	right	left	right
Medianus sensory		--		--		
3rd dig. wrist						
Peroneus superficial sensory	3.11	--	1.40	--	45.0	
Lat leg – dorsum of foot						
Radialis sensory		2.15		7.8		46.5
Forearm-thumb						
Suralis sensory	--	--	--	--		
Mid-calf ankle						

**Table 3:** Motor nerve conduction study ADM: abductor digiti minimi; EDB: extensor digitorum brevis

Motor	Latency (ms)		Amplitude (mV)		Conduction velocity (m/s)		F-latency (ms)	
	left	right	left	right	left	right	left	right
Medianus motor								
Wrist-APB		5.71		1.88				32.7
Elbow-wrist		10.7		1.60		42.1		
Peroneus muscle								
Ankle-EDB	6.67	6.06	0.14	0.078			--	
Fib.head-ankle	13.6		0.10		37.5			
Tibialis motor								
Ankle-abd hal	--	--	--	--				
Ulnaris motor								
Wrist-ADM		2.71		6.9				33.1
Bl. Elbow-wrist		7.71		6.0		44.0		

Following these findings, an autoimmune screen, paraneoplastic screen, viral serology and hemoglobin A1c testing were arranged. Serology was positive for EBV IgG and Varicella IgG. However, she had no signs of an acute viral infection. The HbA1c was raised at 78mmol/mol. All other laboratory testing was unremarkable. The patient had an improving HbA1c compared to her previous readings over the year. Other routine lab tests such as full blood count, urea and electrolytes with liver function tests were within normal range. 

During this patient’s hospital stay, she noted mild improvement in her symptoms with physical therapy and analgesia. The patient was commenced on a low dose of gabapentin which was steadily titrated up to 300mg three times a day. She began to regain her mobility and an assessment of her muscle strength before discharge is as follows: hip flexion right 4/5, left 5/5; hip extension right 4/5, left 5/5; hip abduction right 5/5, left 5/5; hip adduction right 5/5, left 5/5; knee flexion right 4/5, left 5/5; knee extension right 4/5, left 5/5; ankle dorsiflexion 4/5, left 5/5. The patient was noted to have a downtrending HbA1c over the year (Table 4). During her hospital admission, there was no imminent need to adjust the patient's anti-diabetic regimen. She was discharged home with ongoing physiotherapy and an outpatient neurology follow-up appointment. 

**Table 4:** Haemoglobin A1c and glycemic control

Date	Haemoglobin A1c
29/10/2024	48 mmol/mol
08/08/2024	78 mmol/mol
17/07/2024	95 mmol/mol
17/01/2024	94 mmol/mol
25/07/2023	92 mmol/mol
24/03/2023	127 mmol/mol

## Discussion

Diabetic lumbosacral radiculoplexus neuropathy is an uncommon neuromuscular disorder which is often self-limiting but may present a diagnostic challenge due to its wide differential diagnosis and atypical presentation upon initial assessment. Falls constitute a common cause of death, morbidity, mortality, and institutionalization in the elderly. In the UK, one in three adults over 65 experiences at least one fall in a year [4]. There is a wide range of mechanisms responsible for patient presentation with falls which may include but are not exclusive to nutritional, psychological, postural, systemic, or musculoskeletal conditions [4]. 

Many patients may present with any combination of various conditions in the causation of falls. Hence, careful history and clinical examination are vital in establishing the diagnosis. It is also important to rule out other probable causes of plexopathy before diagnosing diabetic lumbosacral radiculoplexus neuropathy. Further investigations such as imaging, laboratory work-up and NCSs are often necessary to guide the diagnosis [3].

Lumbosacral plexus neuropathy is associated with histopathological evidence of ischemic injury and microscopic vasculitis [5]. Therefore, some of the literature may consider this as an inflammatory disorder and state the case for immunomodulatory therapy in the management of such patients [6]. The patient presented in this case had no history of prior malignancy. She had cervical intraepithelial neoplasia, which was excised and had been under regular follow-up, with no evidence of recurrence. A CT scan of her thorax, abdomen, and pelvis and an MRI of the whole spine were unremarkable. 

Although, the patient’s chief complaint was proximal asymmetric lower limb weakness, neurological exam findings revealed evidence of sensorimotor disease with decreased ankle and knee reflexes. Chronic inflammatory demyelinating polyneuropathy (CIDP) was unlikely, given the asymmetric nature of the patient’s disease presentation and nerve conduction findings. Although both pathologies are associated with axonal damage, CIDP is often associated with absent F waves, prolonged distal duration, and abnormal temporal dispersion in NCSs [7]. 

Viruses are a rare cause of lumbosacral plexopathy. Varicella zoster virus (VZV) has been documented to mimic spondyloarthropathy while also causing symptoms akin to brachial and lumbosacral plexopathies [8]. VZV plexopathy has been reported to present with a healing vesicular rash extending across affected dermatomes [8]. However, the patient presented in this case was only positive for VZV IgG. The physical exam did not yield any appearance of a vesicular rash. EBV IgG was also positive. However, physical examination revealed no signs of active EBV infection. The patient denied symptoms of a viral prodrome. The above findings render a viral aetiology to be unlikely. 

Patients with diabetic lumbosacral plexopathy may initially present with debilitating proximal lower limb pain followed by weakness and weight loss [9]. The patient in this case was shown to have an improving HbA1c with likewise trending glycemic control in the months before her hospital admission (Table 4). Plexopathy was the patient’s first presentation with diabetic peripheral neuropathy [10]. Up to the time of writing, the patient had no autonomic symptoms. 

MRI may show increased signal intensity of the muscles and lumbosacral plexus [2]. Nevertheless, NCS/EMG is often pivotal in differentiating between myositis and neuropathy [11]. Note that the patient presented in this report had normal signal intensity of MRI with no evidence of radiculopathy. Inconclusive imaging obviated the need for clinical neurophysiological studies. NCSs in this case were consistent with active widespread peripheral neuropathy and a superimposed lumbosacral plexopathy targeting the affected muscle groups which led to this patient’s lower limb weakness and falls. 

A lumbar puncture was considered by the neurology team. However, the patient began to experience improvement in her symptoms, raising suspicion of diabetes as the aetiology given the monophasic course of her disease progression. In the context of an improving HbA1c with no other concerning symptomology involving other organ systems as well as the absence of systemic symptoms, it was deemed that the most probable cause of the patient’s initial presentation was indeed diabetes. There is no unambiguous evidence about the role of immunomodulatory therapies. Studies are limited due to inadequate sample size and the absence of a control group to compare the progression of recovery [12]. Immunomodulatory treatments such as steroids and IVIG may be considered in severe cases [12,13]. Management of neuropathic pain and physical therapy remain the mainstay of treatment [2]. 

## Conclusions

Diabetic lumbosacral plexopathy constitutes a part of the diabetic neuropathy spectrum. It is a rare long-term complication of diabetes mellitus with a wide differential. Hence, it is pertinent to exclude other causes of similar presentation. Laboratory, imaging, and neurophysiological investigations are necessary for this effect. Physical exam findings are invaluable in establishing a diagnosis. Patients tend to report improvement in their symptoms over time. Management of neuropathic pain may be indicated. Immunomodulatory therapy can also be considered. Neuropathy should always be considered when assessing patients with falls. The case discussed further emphasizes the multidisciplinary team's role in patient care as physiotherapy is pertinent to patient long-term outcomes and quality of life. 
